# Molecular Evolution of RAMOSA1 (RA1) in Land Plants

**DOI:** 10.3390/biom14050550

**Published:** 2024-05-03

**Authors:** Carolina Bellino, Fernando E. Herrera, Daniel Rodrigues, A. Sergio Garay, Sofía V. Huck, Renata Reinheimer

**Affiliations:** 1 Fellow of Consejo Nacional de Investigaciones Científicas y Técnicas de la República Argentina (CONICET), Instituto de Agrobiotecnología del Litoral, Universidad Nacional del Litoral, CONICET, CCT-Santa Fe, Ruta Nacional N° 168 Km 0, s/n, Paraje el Pozo, Santa Fe S3000, Argentina; cbellino1993@gmail.com; 2 Member of Consejo Nacional de Investigaciones Científicas y Técnicas de la República Argentina (CONICET), Facultad de Bioquímica y Ciencias Biológicas, Universidad Nacional del Litoral, Ciudad Universitaria, Paraje El Pozo, Santa Fe S3000, Argentina; herrerafer@gmail.com (F.E.H.); dr.daniel.rodrigues@gmail.com (D.R.); 3 Facultad de Bioquímica y Ciencias Biológicas, Universidad Nacional del Litoral, Ciudad Universitaria, Paraje El Pozo, Santa Fe S3000, Argentina; sergio.alberto.garay@gmail.com; 4 Fellow of Agencia Nacional de Promoción de la Investigación, el Desarrollo Tecnológico y la Innovación, Instituto de Agrobiotecnología del Litoral, Universidad Nacional del Litoral, CONICET, CCT-Santa Fe, Ruta Nacional N° 168 Km 0, s/n, Paraje el Pozo, Santa Fe S3000, Argentina; svictoria.huck98@gmail.com; 5 Member of Consejo Nacional de Investigaciones Científicas y Técnicas de la República Argentina (CONICET), Instituto de Agrobiotecnología del Litoral, Universidad Nacional del Litoral, FCA, CONICET, CCT-Santa Fe, Ruta Nacional N° 168 Km 0, s/n, Paraje el Pozo, Santa Fe S3000, Argentina

**Keywords:** zinc finger, embryophyte, phylogeny, paralogs, collinearity, nuclear localization, secondary structure, divergent motifs, promoter

## Abstract

RAMOSA1 (RA1) is a Cys2-His2-type (C2H2) zinc finger transcription factor that controls plant meristem fate and identity and has played an important role in maize domestication. Despite its importance, the origin of RA1 is unknown, and the evolution in plants is only partially understood. In this paper, we present a well-resolved phylogeny based on 73 amino acid sequences from 48 embryophyte species. The recovered tree topology indicates that, during grass evolution, RA1 arose from two consecutive SUPERMAN duplications, resulting in three distinct grass sequence lineages: RA1-like A, RA1-like B, and RA1; however, most of these copies have unknown functions. Our findings indicate that RA1 and RA1-like play roles in the nucleus despite lacking a traditional nuclear localization signal. Here, we report that copies diversified their coding region and, with it, their protein structure, suggesting different patterns of DNA binding and protein–protein interaction. In addition, each of the retained copies diversified regulatory elements along their promoter regions, indicating differences in their upstream regulation. Taken together, the evidence indicates that the *RA1* and *RA1-like* gene families in grasses underwent subfunctionalization and neofunctionalization enabled by gene duplication.

## 1. Introduction

RAMOSA1 (RA1) is a C2H2 zinc finger protein first cloned and studied in *Zea mays* (maize), a major crop species [1,2]. The RA1 is a small protein (175 amino acids) that is localized in the nucleus despite lacking a traditional nuclear localization signal [3]. The coding sequence of RA1 is characterized by having a unique zinc finger domain and two well-characterized ethylene-responsive element binding factor-associated amphiphilic repression (EAR) motifs towards the C-terminal. In *ra1* mutants, long indeterminate branches replace short determinate branches in both the male and female maize inflorescences, suggesting that RA1 controls meristem fate and identity. Protein–protein interaction in yeast showed that RA1 interacts with RAMOSA1 ENHANCER LOCUS2 (REL2), a co-repressor homolog of *Arabidopsis thaliana* (Arabidopsis) TOPLESS (TPL), to repress the expression of target genes [4]. Indeed, it is well documented that RA1 and REL2 interact in vitro and in vivo via the two EAR motifs of RA1 with the Lissencephaly type1-like homology (LISH/CTHL) region of REL2 [4]. Although RA1 was originally suggested as a strong repressor of transcription, chromatin immunoprecipitation (CHIP-seq) experiments in maize showed that it acts primarily to promote gene expression and that it controls several regulatory and developmental pathways [5]. In addition, recent studies suggest that *cis*-acting regulatory elements upstream of *RA1* are a key factor in modulating branch meristem determinacy affecting ear and tassel morphology in maize and grass inflorescence architecture [6]. Indeed, previous studies have found evidence that *RA1* was a selected locus during maize domestication [7].

So far, the origin and evolution of RA1 in plants is uncertain. In terms of sequence similarity, RA1 is similar to the Arabidopsis SUPERMAN (SUP) protein [2,8]. Within the functional context, SUP is involved during floral development, preventing the initiation of supernumerary stamens, while RA1 has a central role in inflorescence development and does not seem to intervene in floral development [2,8]. The overexpression of *RA1* (*35S:RA1*) in Arabidopsis *sup5* mutants fails to restore the number of stamens in the flower [9]. Likewise, the *35S:RA1* transgenic plants of Arabidopsis generate pleiotropic effects in the plant, such as an increase in the size of the reproductive organs due to cell expansion [10]. These results indicate that the role of RA1 differs from SUP. In particular, within grasses, RA1 was cloned in maize and its closest relatives, but it is absent in the genomes of other grass species of the BOP clade (such as rice, wheat, and oat) [1,11].

Given the (1) importance of RA1 during maize inflorescence development and domestication, (2) its central role as a regulator of various plant development pathways, and (3) the fragmentary knowledge that exists about the evolution and role of the RA1 in other cereals, we decided to study RA1 to deepen our understanding of the protein evolutionary history. For that, we reconstructed the phylogeny of RA1 in embryophytes and identified homologs and paralogs protein sequences. Additionally, phylogeny reconstruction results were analyzed considering genome and chromosome collinearity. Homologs and paralogs of RA1 sequences were comparatively characterized with a focus on conserved/divergent motifs along coding and promoter regions, subcellular protein localization, protein secondary structure, and conformational plasticity. We found a complex pattern of gene duplication followed by different patterns of gene copy retention and losses. Retained copies showed partial conservation of the coding sequences and secondary structure as well as *cis*-elements in the promoter regions, suggesting diversification of protein functionality and upstream regulation. The resulting information will be useful to further explore the mode of action of these proteins in the future. 

## 2. Materials and Methods

### 2.1. Data Sets

Protein sequences were retrieved from Phytozome Database v13 [12], National Center for Biotechnology Information [13] and Gramene Released 66 [14] in March 2023 (Datasheet 1 available at Mendeley Digital Repository, https://doi: 10.17632/m45fk8hxs4.1, accessed on 8 February 2024, Table S1). Three annotated protein sequences of *A. thaliana* (SUP, AT3G23130), *Setaria viridis* (XP_034583085), and *Z. mays* (NP_001143449) were used as templates for a BLASTp search in each of the 50 studied embryophyte genomes: *Amborella trichopoda* (AmTr), *Anana comosus* (Ac), *Aquilegia coerulea* (AqCo), *A. thaliana* (At), *Brachypodium distachyon* (Bd), *Brachypodium sylvaticum* (Bs), *Brachypodium stacei* (BrSt), *Brassica rapa* (Br), *Carica papaya* (Cp), *Capsella rubella* (Cr), *Cenchrus americanus* (Ca), *Ceratodon purpureus* (CePu), *Citrus clementina* (Cc), *Dioscorea alata* (Da), *Eleusine coracana* (Ec), *Hordeum vulgare* (Hv), *Joinvillea ascendens* (Ja), *Leersia perrieri* (Lp), *Marchantia polymorpha* (Mp), *Medicago truncatula* (Mt), *Miscanthus sinensis* (Ms), *Olea europaea* (Oe), *Oryza barthii* (OrBa), *Oryza brachyantha* (Ob), *Oryza glaberrima* (Og), *Oryza glumipatula* (OrGl), *Oryza meridionalis* (Om), *Oryza nivara* (On), *Oryza punctata* (Op), *Oryza rufipogon* (Or), *Oryza sativa* (rice) (Os), *Panicum hallii* (Ph), *Panicum virgatum* (Pv), *Pharus latifolius* (Pl), *Physcomitrella patens* (Pp), *Populus trichocarpa* (Pt), *Prunus persica* (PrPe), *Ricinus communis* (Rc), *Selaginella moellendorffii* (Sm), *Setaria viridis* (Sv), *Sorghum bicolor* (Sb), *Solanum tuberosum* (St), *Theobroma cacao* (Tc), *Thinopyrum intermedium* (Ti), *Thuja plicata* (Tp), *Urochloa fusca* (Uf), *Vaccinium darrowii* (Vd), *Zea luxurians* (Zl), *Z. mays* (Zm), and *Zizania palustris* (Zp). Duplicated sequences were pruned, and raw datasets were scanned using Multiple Expectation maximizations for Motif Elicitation (MEME) search with MEME v5.5.2 [15]. The search was set up to a maximum of 15 motifs and default settings. Based on MEME exploratory scans, sequences containing an incomplete zinc finger motif and/or truncated C-terminal EAR motif were discarded. Protein sequences were aligned with the online version of MAFFT using an E-INS-i strategy [16]. The alignments were manually inspected using MEGA v.10.2.4 [17]. The final aligned dataset containing a total of 73 sequences is presented in Figure S1. 

### 2.2. Phylogeny

Phylogenetic trees were built with MrBayes 3.2.6 [18] via the CIPRES Science Gateway [19] using a fixed substitution model and run for 15 million generations (sampling every 1000 generations). Four Markov chains were run simultaneously in two independent runs, starting with a random tree until the convergence diagnostic (standard deviation of split sequences) dropped below 0.01. The first 3.75 million generations were discarded as burn-in (25%), and the rest of the samples from the two replicates were combined. The convergence and the effective sample size (>100) of each replicate were checked using Tracer v1.7.2 [20]. A majority rule consensus of 22,502 trees was constructed and visualized using Mesquite [21]. Further tree edition was performed using inkscape v1.2.2. To search for chromosomal collinearity (synteny) among duplicated genes, we used the GENESPACE Synteny Viewer available at Phytozome Database v13 [12,22] using *A. thaliana* TAIR10, *O. sativa* v7.0, *S. viridis* v2.1, *Z. mays* Zm-B73-REFERENCE-NAM-5.0.55, and *Z. mays* RefGen_V4 as reference genomes.

### 2.3. Conserved Domain Analysis

To identify conserved motifs, sequences from 16 grass species (of 27) were selected to have, at least, one representatives of each studied genus. In total, 35 amino acid sequences were scanned with MEME using MEME v5.5.1 [23]. Based on the E-values cut-off (greater than 0.01), the search was set to 15 domains ranging from 6 to 50 amino acids with default settings after preliminary optimization runs. For each lineage, a box scheme was presented graphically to show the distribution and frequency of occurrence of their specific motifs.

### 2.4. Subcellular Localization of RA1 and RA1-Like Proteins

#### 2.4.1. Plant Growth Conditions 

*Nicotiana benthamiana* (tobacco) seeds were grown directly on soil at 22–24 °C and 120 µE (µEinstein = µmol m^−2^ s^−1^) in a growth chamber under long-day conditions (16 h of light and 8 h of darkness). *Setaria viridis* accession A10 seeds were grown in a growth chamber at 24–28 °C and 300 µE under long-day conditions. *Zea mays* cultivar B73 seeds were grown in a greenhouse (Instituto de Agrobiotecnología del Litoral) at 25–28 °C and 800–1000 µE under long-day conditions. The wild-type and transgenic seeds used in this work are deposited at the CRBIAL biological repository of the Instituto de Agrobiotecnología del Litoral (Santa Fe, Argentina). 

#### 2.4.2. Genetic Constructs

The open reading frame of *RA1 homologs* from *Z. mays* and *S. viridis* and paralogs of *S. viridis* were amplified by PCR using Taq Pegasus DNA polymerase (Productos Bio-Lógicos PBL SA, Buenos Aires, Argentina) with specific primers carrying restriction sites (Table S2). Amplicons were digested with the corresponding enzymes (Promega Corporation, Madison, WI, USA) and ligated into the entry vector pENTR3C (Invitrogen) using T4 DNA ligase (Invitrogen, Carlsbad, CA, USA). For the subcellular localization analyses, each sequence-verified entry vector clone was sub-cloned into the pFK247 destination vector by site-specific recombination using Gateway LR Clonase II Enzyme Mix (Invitrogen) [24]. The pFK247 vector is derived from the pGreen vector series in which a 35S CaMV promoter drives the expression of an N-terminal fusion protein with Green Fluorescent Protein (GFP) [25].

#### 2.4.3. Transient Transformation

Tobacco leaves were agro-infiltrated with *Agrobacterium tumefaciens* LBA4404 cells carrying the appropriate translational fusion construct described above. In order to suppress gene silencing, *A. tumefaciens* cells that express the tomato bushy stunt virus p19 protein [26] were used in the co-infiltration method, as previously reported by Belda-Palazón et al. [27]. For that, an equal mixture of Agrobacterium strains containing the appropriate translational fusion constructs and the p19 plasmid was prepared for co-infiltration. For transient transformation, the abaxial side of four-week-old tobacco leaves were co-infiltrated with a needleless syringe, as previously described [28]. After infiltration, the plants were exposed to light for one hour and moved into a dark chamber overnight. Seventy-two hours after infiltration, samples were collected at the beginning of the photoperiod and used for visualization under confocal laser scanning microscopy. Fluorescence of at least two transformed leaves from three plants of similar age was analyzed with confocal microscope. The experiments were repeated at least two times for each construct.

#### 2.4.4. Nuclei Staining

The reagent 4′,6-diamidino-2-phenylindole (DAPI; Sigma, St. Louis, MO, USA) was used to stain the nuclei. Before fluorescence confocal microscopy analysis, the agro-infiltrated tobacco leaves were removed from the plant and cut into one-inch-diameter circles; these were incubated in distilled water supplemented with 1µg/mL DAPI for 5 to 45 min. Then, leaf sections were mounted on a microscope slide and covered with distilled water for observation through the leaf abaxial side.

#### 2.4.5. Fluorescence Microscopy

Digitized confocal images were acquired at 1024 × 1024 pixel resolution with a ×20 dry objective on a TCS ST8 inverted confocal laser scanning microscope (Leica Microsystems GmbH) by using a 488 nm excitation line laser for GFP, and spectral detection was made at 497 and 537 nm for GFP. For DAPI laser excitation, the wavelength was set to 405 nm, and detection was carried out at 410–492 nm. Image plates were assembled with inkscape v1.2.2. 

### 2.5. Molecular Structure 

Molecular structure predictions were carried out using the PsiPred Workbench (http://bioinf.cs.ucl.ac.uk/psipred, accessed on 4 October 2023). Predictions on secondary structure we carried out with PsiPred 4.0 tool [29]. Information on residues that can be predicted as disordered and that are structured by joining other proteins were extracted using DisoPred 3.1 tool [30].

### 2.6. Molecular Dynamic Simulations

Molecular dynamics studies in solution were carried out using the GROMACS 2021.5 package [31] in conjunction with the AMBER99SB-ILDN force field [32]. The molecular dynamics protocol was performed for a fragment peptide that comprises the zinc finger domain regions of SUP (aa: 42–78), which was used in the Nuclear Magnetic Resonance (NMR) structure determination [33]. Indeed, this fragment was chosen from a multiple sequence alignment of plant QALGGH proteins that contains only a single zinc finger domain, most of them from *A. thaliana* [33]. A multiple sequence alignment of SUP and RA1 proteins identifies the fragment of RA1 (aa: 32–77) as the one corresponding to the peptide from SUP used in the NMR experimental determination. Since experimental structure determination of the RA1 zinc finger region is not available, the initial structure of the RA1 fragment peptide used for starting the molecular dynamics simulation was taken from the AlphaFold best scored model of the complete protein [34]. The AlphaFold server assigns a high level of confidence to the predicted structure of the RA1 zinc finger region. Values of the predicted local distance difference test (pLDDT), which are used to assess the confidence level, are higher than 90/100 for residues from Y46 to H68 and higher than 75/100 for residues from R69 to H83. This initial model has a helix feature spreading beyond the RA1 fragment limits obtained from the multiple sequence alignment; therefore, we decided to use an extended fragment of RA1 (aa: 32–83) for the peptide simulation to avoid disturbing this secondary structure element.

The simulation protocol was the same for each simulation, and it consisted of (1) total energy minimization of the initial system using the steepest descent energy minimization algorithm and a tolerance of 100 kJ/mol; (2) molecular dynamic simulations with position restraint of the protein heavy atoms for 2 ns in order to allow the water molecules to rearrange around the protein; (3) simulated annealing with position restraint of the zinc finger heavy atoms, increasing the temperature from 300 K to 400 K during 200 ps, equilibrating the temperature for 600 ps, and finally gradually cooling the system back to 300 K during 4.2 ns (5 ns in total) in order to enhance conformational sampling; and (4) molecular dynamics production run at a temperature of 300 K for 1 μs. In all cases, the proteins were inserted into an octahedral box of simple point charge (SPC) water molecules. The minimum distance between the protein and the simulation box was 1.1 nm, to avoid any inappropriate behavior of the water molecules. The timestep of 2 fs was used for the integration of the equations of motions. The Berendsen thermostat and barostat were used to couple the systems to a temperature of 300 K (with coupling time constant 0.1 ps) and a pressure of 1 bar (with coupling time constant 2.0 ps) baths, respectively. The particle mesh Ewald method [35] was used to treat long-range Coulombic interactions. The LINCS algorithm [36] was used to constrain bond lengths of protein atoms and SETTLE for the water molecules [37]. Van der Waals forces were considered up to distances of 1.2 nm, and Coulomb interactions were truncated at 1.2 nm. The root-mean-square deviation (RMSD) of backbone atoms was calculated over the 1μs simulated time, while the root-mean-square fluctuation (RMSF) was computed using the final 200 ns of the molecular dynamic’s trajectory. Both RMSD and RMSF were calculated using the central structure as the reference for rigid fitting and distance calculations. This central structure is determined from the RMSD matrix in a preliminary calculation performed using as reference the initial structure of the simulation, as it is the one with the lowest average RMSD to all the structures within the equilibrated time interval after the fitting procedure (see gmx cluster module in ref. [31]). This central structure is significant since it is representative of the geometry of the peptide in equilibrium.

### 2.7. Analysis of Promoter cis-Acting Regulatory Elements in Grasses

To identify conserved regulatory motifs on the promoter regions, we downloaded 2 kb upstream genomic sequences from the translation initiation site of *RA1*, *RA1-like A*, and *RA1-like B* of 16 grass species. Downloaded sequences were screened to search for codified genes using FGENESH (http://www.softberry.com/berry.phtml, ccessed on 10 August 2023) [38]. When additional genes were identified, retrieved sequences were shortened accordingly. Retrieved sequences were organized in eight different datasets: (a) RA1 Andropogoneae dataset, comprising *RA1* promoter sequences from four Andropogoneae species; (b) RA1 Paniceae dataset, comprising *RA1* promoter sequences from four Paniceae species; (c) RA1-like A Andropogoneae dataset, comprising *RA1-like A* promoter sequences from three Andropogoneae species; (d) RA1-like A Paniceae dataset, comprising *RA1-like A* promoter sequences from four Paniceae species; (e) RA1-like A BOP dataset, comprising *RA1-like A* promoter sequences from five species of BOP clade; (f) RA1-like B Andropogoneae dataset, comprising *RA1-like B* promoter sequences from three Andropogoneae species; (g) RA1-like B Paniceae dataset, comprising *RA1-like B* promoter sequences from five Paniceae species; and (h) RA1-like B BOP dataset, comprising *RA1-like B* promoter sequences from six species of BOP clade. Potential *cis*-acting regulatory elements were determined against the JASPAR CORE 2022 database of nonredundant transcription binding profiles from plants [39] using XSTREME [40]. Each dataset was scanned for motifs ranging from 6 to 30 characters and default settings. We considered motifs with E-value < 0.05. 

## 3. Results

### 3.1. Phylogenetic Analysis

#### 3.1.1. SUP Evolution and the Origin of RA1

**Figure 1:**
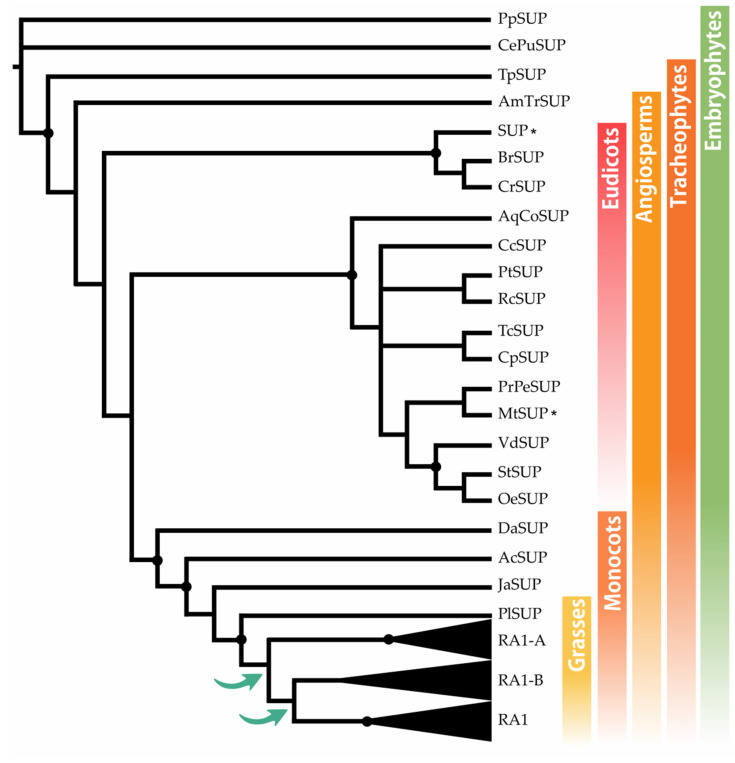
SUP evolution and the origin of RA1. Majority rule consensus tree (N = 22502 trees) of RA1 transcription factor in embryophytes generated by Bayesian inference using 73 peptide sequences (Figure S1 and Table S1). Black dots indicate Bayesian posterior probability (PP) = [0.9 to 1]. Green arrows indicate gene duplication events. Black asterisk points out proteins with known function (Table S2).

The BLASTp searches recovered SUP sequences along most of the embryophyte species, except from *M. polymorpha* and *S. moellendorffii*. To identify the origin of RA1 and understand its evolution, we reconstructed a phylogenetic tree with amino acid sequences obtained from genomes of grasses and other embryophytes. For that, we used SUP sequences from non-seeds plants (*P. patens* and *C. purpureus*) as outgroups. The recovered tree topology, and the presence of a single copy sequence of the grass *P. latifolia* suggests that RA1 originated from two successive duplications of SUP that occurred at the split of the BOP and PACMAD clade (Figure 1). As a result of these duplications, three lineages of sequences were generated; one of them includes RA1 sequences, and the other two are here named arbitrarily RA1-like A and RA1-like B. In this tree, the evolutionary relationships among RA1 and RA1-like remain unclear.

#### 3.1.2. Evolution of RA1 in Grasses

**Figure 2:**
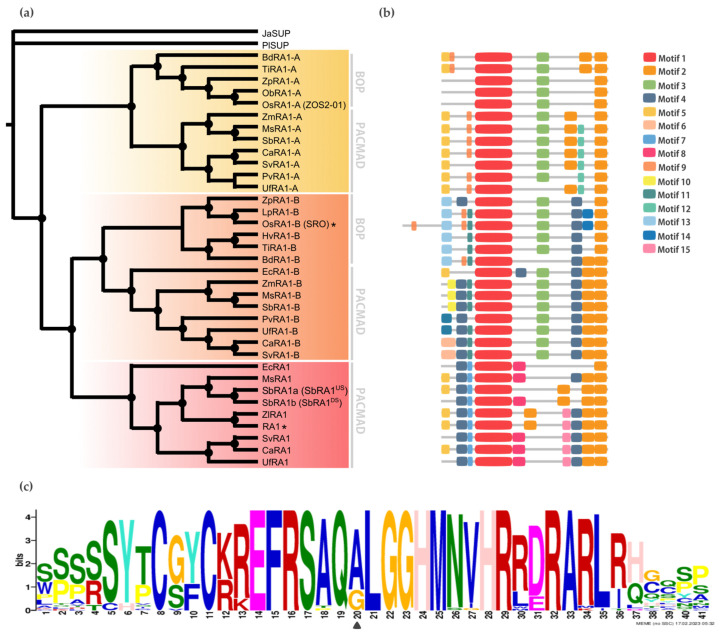
Evolution of RA1 in grasses. (**a**) Majority rule consensus tree (N = 22502 trees) of RA1 and RA1-like transcription factors in grass species generated by Bayesian inference using 35 amino acid sequences (Figures S2 and S3 and Table S1). Each clade is identified by a color. Black dots indicate Bayesian posterior probability (PP) = [0.9 to 1]. Black asterisk points out proteins with known function (Table S3). (**b**) Motif distribution patterns on RA1 and RA1-like amino acid sequences in grass species. Colored boxes represent motif occurrence (Figures S7 and S8). (**c**) Sequence representation logo of Motif 1 obtained from the multiple sequence alignment (Figure S9). Black arrowhead indicates the amino acid variant (A- > G) in Motif 1.

To gain resolution on the relationship between RA1 and RA1-like, we reconstructed a new phylogeny with amino acid sequences obtained from 16 genomes of grass species using as an outgroup the SUP sequence from the monocot *J. ascendence* (Figure 2a, Figures S2, and S3). The obtained tree topology suggests the possibility that (1) RA1 was originated after a second duplication around the split of the BOP and PACMAD clades, (2) RA1 is sister to the RA1-like B proteins, (3) RA1 was lost in the BOP clade and currently is present in the PACMAD clade, (4) RA1-like A and RA1-like B are found in species of both BOP and PACMAD clades, and (5) species-specific duplications and loss of some copies of RA1 and RA1-like suggest a complex evolution of these molecules during the diversification of the grasses (Figure 2a and Figure S4). Overall, *SUP*, *RA1*, and *RA1-like* are single-copy genes, except for the two *RA1* variants found in *S. bicolor*, as previously described [2] (Figure 2a).

Genome annotations indicate that *SUP* is on Chr3:8242256…8243372 of the Arabidopsis genome, whereas *RA1*, *RA1-like A,* and *RA1-like B* are on maize chromosomes Chr7:114958642…114959398, Chr5:68312034…68312578, and Chr10:103427449…103428364, respectively. Chromosomal collinearity visualization maps indicate that Arabidopsis chromosome 3 lacks synteny with maize chromosomes 7, 5, and 10 (Figure S5). In contrast, *RA1* and *RA1-like* genes are placed in syntenic chromosome blocks among maize, Setaria, and rice (Figure S6). These results suggest that RA1 and RA1-like are syntenic paralogs. 

### 3.2. Conserved Motifs Analysis among RA1 and RA1-Like Coding Sequences

To discover specific features that characterize the coding region of each lineage, we performed a motif analysis (Figure 2b). We searched for 15 different motifs using MEME to better understand sequence plasticity (Figures S7 and S8). Some of them are lineage-specific (for instance, Motif 7 (WPPPQVRS), Motif 8 (PPPNPNPSCTVLDL), Motif 11 (FPWPPQ), Motif 12 (VVCSCSST), and Motif 13 (MESRSAARAGDQQH)), and others are shared by lineages, such as Motif 3 (ARAPJPNLNYSPPHPA), which is present in RA1-like A and RA1-like B sequences, whereas Motif 4 (APPVVYSFFSLAASA) is shared by RA1 and RA1-like B sequences (Figure 2b and Figure S7). All the sequences have in common the presence of one C2H2 zinc finger domain (Motif 1 (SSSSSYTCGYCKREFRSAQALGGHMNVHRRDRARLRHGQSP)) (Figure 2b and Figure S9). In particular, sequences from RA1 lineage have the variant QGLGGH in the zinc finger domain, as it was previously reported [2] (Figure 2c). In addition, Motif 2 (GDGAEEGLDLELRLG) appeared two to three times towards the C-terminal of all the sequences. Our analysis identified two Motif 2 along the C-terminal of the RA1-like sequences, whereas three Motif 2 were detected on most of the RA1 sequences of the PACMAD clade (Figure 2b and Figure S9). In addition, we found that the number of Leu (L) and the distances between the Motifs 2 vary among clades (Figure 2b and Figure S9). According to the literature, Motif 2 is a putative EAR-like repressor motif [2,41,42,43,44,45].

### 3.3. Subcellular Localization

**Figure 3:**
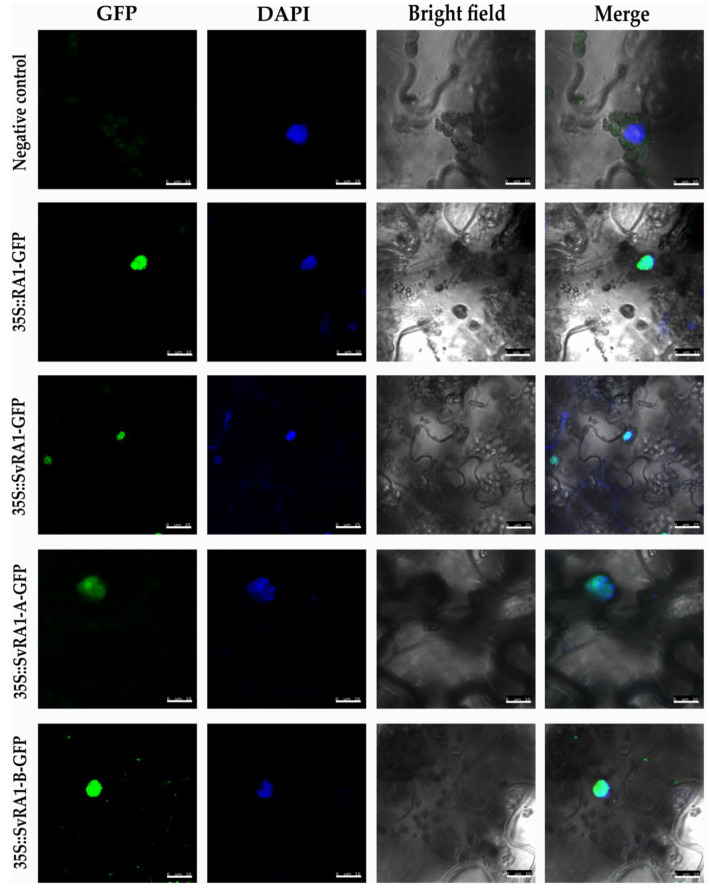
Sub-cellular localization analysis of RA1 and RA1-like proteins. Sub-cellular localization of the RA1-GFP, SvRA1-GFP, SvRA1-like A-GFP, and SvRA1-like B-GFP constructs in tobacco leaf epidermal cells. Green color is GFP protein signal. Blue color represents DAPI-stained nucleus.

To determine the in vivo subcellular localization of the RA1 and RA1-like proteins of maize and *S. viridis*, we generated a N-terminal translational fusion with GFP. The 35S:GFP:RA1 and 35S:GFP:RA1-like constructs were analyzed by a transient expression system in agro-infiltrated young tobacco leaves. The fluorescence was observed through a laser scanning confocal microscope. DAPI staining was used to visualize nuclei localization. As shown in Figure 3, the RA1 and RA1-like proteins are localized in the nuclei of tobacco epidermal cells.

### 3.4. Molecular Modeling

#### 3.4.1. Proteins’ Secondary Structure and Conformational Plasticity

**Figure 4:**
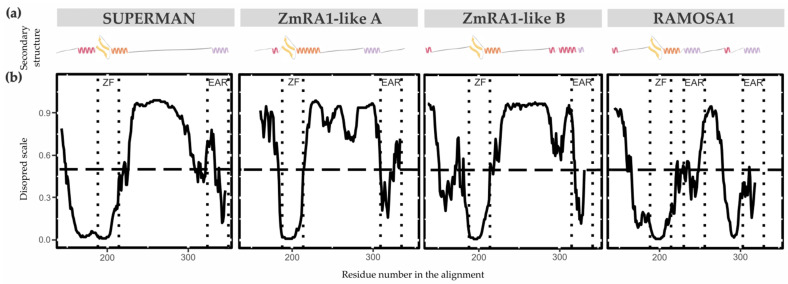
Protein secondary structure and conformational plasticity. (**a**) Secondary structure prediction of SUP, ZmRA1-like A, ZmRA1-like B, and RA1 proteins obtained by PsiPred server. α-helix corresponding to the zinc finger domain is represented in orange. α-helix corresponding to Motif 2, putative EAR motif, is represented in violet. Other α-helices are represented in pink. β-strands are represented in yellow. (**b**) Relative disorder levels of the structures measured in a range of 0 to 1.0 (Figure S10 and Table S4). Levels above 0.5 (dashed line) are considered disordered regions. Abbreviations: ZF, zinc finger domain; EAR, EAR repressor motifs.

The secondary structure properties of the SUP/RA1 members are poorly characterized [33,46]. Only the zinc finger C2H2-type of SUP has had its secondary structure experimentally solved by NMR [33]. So, to gain perspective on protein structure among SUP, RA1, and RA1-like, secondary structures predictions were carried out using the PsiPred Workbench (http://bioinf.cs.ucl.ac.uk/psipred) and its PsiPred 4.0 tool [29]. We also used the server’s tool DisoPred 3.1 [30] to predict regions that cannot be assigned a known secondary structure, which are termed as disordered regions. The same algorithm also predicts regions with high probability of binding other proteins. These properties are graphically shown in Figure 4 and Figure S10 and Table S4 as a function of the residue number/name. Overall, the N-terminal region of SUP, RA1, and RA1-like is predicted as disordered with protein binding affinities. Allocated within the N-terminal disordered segment are common motifs shared between lineages, such as Motif 4 and 5 (Figure 2b). The N-terminal disordered region is followed by a region defined as a coil that continues with the C2H2-type domain. The structural predictions suggest the zinc finger to be formed by a β-sheet (two consecutive β-strands) followed by an α-helix (Table S4). In general, the C-terminal of RA1 and RA1-like is presented as a disordered region with protein binding affinities. Allocated within the C-terminal disordered segment is Motif 3 shared by RA1-like A and RA1-like B (Figure 2b). In addition, we found stabilized ordered segments, such as two to three α-helices. These ordered segments are mostly correlated with the allocation of Motif 2 (Figure 4 and Figure S10 and Table S4).

#### 3.4.2. Zinc Finger Molecular Dynamics Simulation

The zinc finger of SUP (1NJQ) is a structure resolved by NMR (20 models), which has 75% sequence identity with the zinc finger of RA1 (Figure S11a). There is no experimental structure of RA1. Given differences in sequence identity between SUP and RA1, we performed 1μs molecular dynamics simulations of each protein zinc finger domain to evaluate differences in the structural stability of these variants. The RMSDs of these simulations as a function of simulated time (Figure S11b) show small departures from their initial values (average RMSD values lower than 0.5Å). This fact is associated with the robustness of the structure of the zinc finger domain of both proteins and the proximity of the initial models to the equilibrated structures. We assumed that the simulated time in the period [0.8μs, 1.0μs] is adequate to calculate equilibrium properties of the systems since the RMSDs of the simulations of both proteins show small fluctuations without any appreciable definite tendency.

**Figure 5:**
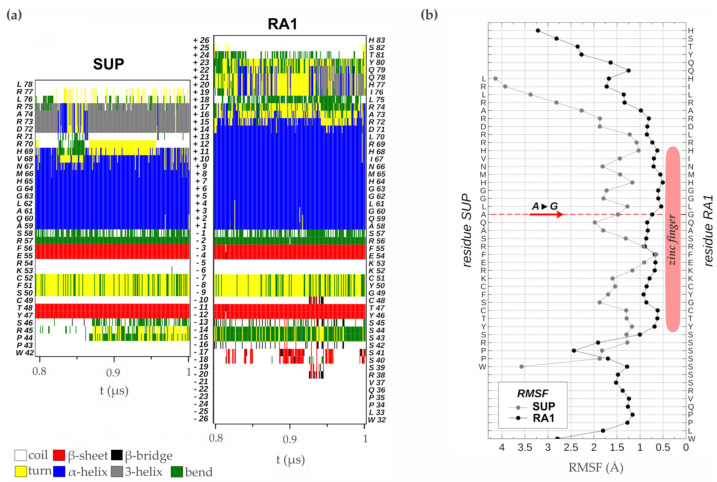
Zinc finger molecular dynamics simulation. (**a**) Time evolution of the secondary structure of SUP and RA1 proteins as determined by the DSSP algorithm using the simulated structures in the thermodynamics equilibrium interval (0.8 to 1.0 μs). At the side of the graph, the amino acid sequences are quoted with their residue number in the proteins. In the center of the graph, the relative position of each residue in reference to the first one of the α-helix N-terminal is indicated. (**b**) RMSF of the backbone atoms of each amino acid sequence in the equilibrium interval (0.8 to 1.0 μs), taking as reference for fitting the structures the one with smallest RMSD in the same period.

Analysis of the time evolution of the secondary structure of SUP and RA1 peptides showed the classical pattern of a zinc finger with a β-sheet composed of two strands (Y47T48-F55E56 and Y46T47-F54E55 in SUP and RA1, respectively) and a turn stabilized by H-bonds (S50F51C52 and G49Y50C51 for SUP and RA1), followed by an α-helix (A59:H69 and A58:R72 for SUP and RA1) (Figure 5a, Table S4). The α-helix structural motif is four residues longer for the case of RA1 (an additional turn). Beyond the α-helix structural motif, the SUP peptide presents a turn of a 310-helix (D72:R75) (Figure 5a, Table S4). We observed an additional structure with fluctuating characteristic 310-helix, α-helix, and turns in RA1 peptide (I76:Y80) (Figure 5a, Table S4). 

Previous work suggests that the change of Ala (A) to Gly (G) in the QALGGH motif of the RA1 zinc finger could lead to an enhanced mobility of the residues in the α-helix [2]. However, such a hypothesis has not been tested yet. Here, we performed a comparative RMSF analysis between RA1 and SUP to test whether the G affects the structure dynamic of the zinc finger α-helix (Figure 5b). The RMSF of the SUP and RA1 amino acids were calculated in the equilibrium interval of the simulations for the backbone atoms of each residue. We found that, at this site, the amino acids of RA1 show smaller fluctuations than the respective amino acids of SUP (Figure 5b). One could argue that the effect of the substitution of A by G may not be local; however, we found that the RA1 amino acids show smaller fluctuations along all the extensions of the canonical zinc finger domain. The existence of a longer α-helix motif in the RA1 peptide, followed by the additional helix pattern, causes the region of small fluctuations or relative rigidity of the peptide to extend well beyond the canonical zinc finger motif (Figure 5b). These results indicate that substituting A for G does not impact the three-dimensional structural mobility at that site, nor does it affect the overall structure of the zinc finger.

### 3.5. Cis-Acting Regulatory Elements Analysis among RA1 and RA1-Like Promoter Sequences

**Figure 6:**
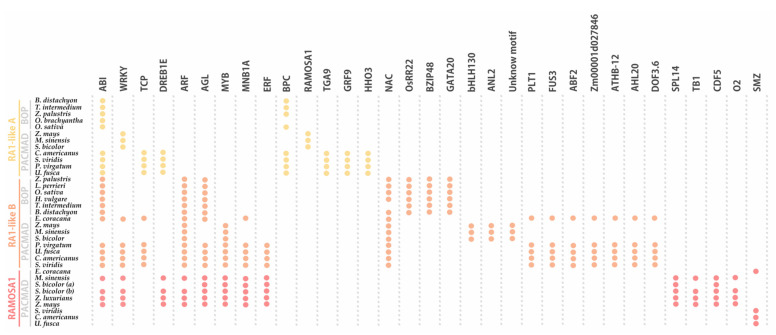
Noncoding *cis*-elements identified in predicted promoter regions of *RA1* and *RA1-like* genes from grass species. Colored circles represent presence of conserved motifs in the promoter. Motif consensus sequences are showed in Table S5.

When the promoter of *RA1* and *RA1-like* genes of 16 grass species were examined, many regulatory motifs were identified and grouped into 33 types of elements, according to the corresponding transcription factor (TF) family (Figure 6, Table S5). Conserved sequences containing binding motifs for well-known transcriptional regulators were recognized. We identified TF motifs involved in different biological processes related to (1) seed germination, such as embryo development, somatic embryogenesis, and positive regulation of cell population proliferation; (2) plant development, such as regulation of secondary shoot formation, leaf development, flower development, and root development; (3) response to abiotic stress, such as response to salt stress, response to light stimulus, and response to water; and (4) response to biotic stress, such as defense response to bacterium (Table S6). In addition, we identified TF binding sites related to positive or negative regulation of hormone signaling pathways, hormone biosynthetic processes, as well as auxin, gibberellin, abscisic acid, and ethylene responses (Table S6).

The conserved regulatory motifs harbored nine putative TF binding sites for RA1-like A lineage, 22 putative TF binding sites for RA1-like B lineage, and 13 putative TF binding sites for RA1 lineage. We found two conserved motifs shared by *RA1-like* and *RA1* promoter sequences: (1) ABI-motifs are present among at least 24 of 35 promoter sequences analyzed; (2) WRKY-motifs were identified in 12 of 35 promoter sequences.

*RA1-like A* shares (1) TCP-motifs with the promoter sequences of *RA1-like B* of the PACMAD clade, except for the ones of Andropogoneae, and (2) DREB1E-motifs with Andropogoneae promoter sequences of *RA1*. 

*RA1-like B* has five motifs in common with *RA1*: (1) ARF-motifs are present in all *RA1-like B* and *RA1* promoter sequences of the Andropogoneae tribe; (2) AGL-motifs are well conserved in *RA1-like B* promoter sequences, except in the Andropogoneae tribe, while AGL-motifs are restricted to the Andropogoneae tribe in RA1 lineage; (3) MYB-motifs were identified in *RA1-like B* sequences of the PACMAD and *RA1* promoter sequences from the Andropogoneae tribe; (4–5) MNB1A-motif and ERF-motifs were identified among *RA1-like B* promoter sequences of the PACMAD clade, except for the ones of Andropogoneae, and *RA1* promoter sequences from the Andropogoneae tribe.

We found that each species lineage copy is characterized by unique *cis*-elements. The RA1-like A lineage has conserved BPC-motifs outside the Andropogoneae tribe and a RA1 binding motif in the Andropogoneae tribe. TGA9-motif, GRP9-motif, and HHO3-motif were identified only in promoter sequences from PACMAD clade species outside the Andropogoneae tribe. The RA1-like B lineage is characterized by the presence of NAC-motifs on all promoter sequences analyzed, except for the one of *T. intermedium*. OsRR22-motif, BZIP48-motif, and GATA20-motif are restricted to the BOP clade. Between species from the PACMAD clade, bHLH130-motif, ANL2-motif, and an unknown motif were identified in the Andropogoneae tribe, while PLT1-motif, FUS3-motif, ABF2-motif, Zm00001d027846-motif, ATHB-12-motif, AHL20-motif, and DOF3.6-motif were identified outside the Andropogoneae tribe. Finally, the promoter sequences of the RA1 lineage are characterized by the presence of (1) SPL14-motif, TB1-motif, CDF5-motif, and O2-motif sequences in the Andropogoneae tribe and (2) SMZ-motif outside the Andropogoneae tribe. 

## 4. Discussion

RAMOSA1 is a small C2H2 zinc finger transcription factor that has an unquestionable role during maize inflorescence development and domestication [1,4,5]. Indeed, RA1 is the core of the RAMOSA circuit that regulates the fate of axillary meristem determining the final form of the maize inflorescence architecture. Despite its remarkable role, so far, the origin and the evolution of RA1 has remained uncertain. It has been mentioned that SUP may be the Arabidopsis homolog of RA1 based on sequence similarity; however, it has been documented that SUP and RA1 have different functions [1,8,47]. 

We identified that SUP was present, at least, from the early diversification of embryophytes (land plants). Based on such results we reconstructed the evolutionary history of SUP/RA1 in embryophytes. The phylogeny presented here suggests that RA1 arose from two successive duplications of SUP occurred at the diversification of the BOP and PACMAD clades. The first duplication gave rise to the RA1-like A lineage that is sister to the paralogs RA1-like B and RA1 lineages. The phylogeny is also supported here by genome synteny studies. Indeed, the previous literature and the collinearity analysis presented in this work suggest that both duplications may correlate with GD that occurred at the base of BOP/PACMAD separation (at ~80 million years ago) [48,49,50]. Interestingly, *RA1* and *RA1-like* paralogs genes are exclusive of grasses and show different patterns of retentions and losses, as is usually observed after genome duplication events [48,49,50]. It is well documented that differential gene loss or subfunctionalization and neofunctionalization of retained copies, as the case presented here, may promote morphological, physiological, and ecological diversification, in particular, in grasses [51,52].

The nuclear localization of RA1 is well described [3]; however, the localization of RA1-like proteins was unknown. To generate knowledge on the general role of such proteins, we performed protein subcellular localization studies. We observed that RA1 and RA1-like localized in the nucleus besides lacking a classical nuclear localization signal [3]. So far, our results indicate that RA1 and RA1-like proteins may move to the nucleus to regulate gene expression like most of the C2H2 zinc finger proteins. 

### 4.1. Duplication of SUP/RA1 Correlates with Changes in the Coding Region, Secondary Structure, and Diversification of the Binding Properties of Their Promoter Regions

#### 4.1.1. Evolution of RA1 and RA1-Like Amino Acid Sequences

We found conserved and divergent motifs along RA1 and RA1-like amino acid sequences. All the sequences studied in this work presented the C2H2-type zinc finger with the motif QALGGH, except for the variant of QGLGGH in the RA1 sequences. Overall, we observed conserved motifs between RA1-like A and RA1-like B and between RA1-like B and RA1 sequences as well as specific motifs that characterize each lineage. Some of these conserved motifs were identified in regions predicted as disordered structures with binding protein affinities, suggesting that RA1 and RA1-like have diversified their function through variations in protein–protein interaction. The functional significance of this observation is still to be determined.

Interestingly, we identified motifs linked to positive and negative regulators of the transcription, as was previously identified in SUP [53]. Negative elements at the N-terminal of the zinc finger domain (Motif 1) might affect nucleosome positioning in the core promoter region, as was shown in SUP [53]. Also, the region around the C-terminal (Motif 2) could be a target site for methylation and silencing the gene expression [53,54]. Likewise, results of ChIP-seq and genome-wide analysis of RA1 occupancy showed that RA1 can repress and also activate genes associated with nucleic acid-related processes, such as chromatin, and TFs implicated in cell specification, with final effects on maize inflorescence development [5]. 

In particular, in the present study, Motif 2 was identified as a putative EAR-like repressor motif (xLxLxLx; [41,43,44,45,55,56,57]). Indeed, SUP has been characterized as a repressor protein whose repression activity falls on the unique C-terminal EAR motif [42]. However, RA1 was described as a repressor protein bearing two EAR motifs: an EAR motif towards the C-terminal of the protein as in SUP [2] and a second one close to the C-terminal of the zinc finger domain [4]. It was documented that both EAR repressor motifs of RA1 are involved in the interaction with REL2 (a co-repressor homolog to TPL of Arabidopsis), which, in turn, regulates their target genes promoting the formation of short branches bearing paired spikelets in maize [4,58]. Similarly, more recently, it has been documented that SUP acts as a repressor of B class genes in the 4th whorl during flower development by interacting with TPL via its single EAR motif located at the C-terminal region of the protein [59]. Indeed, a site-directed mutagenesis of the SUP EAR motif demonstrated that the removal of a Leu will abolish the interaction between SUP and TPL [59]. Interestingly, among RA1 sequences, we found natural Leu mutations in Motif 2 located next to the zinc finger domain in sequences of PACMAD species, excepting the Andropogoneae. In addition, the results presented in this work indicate that most RA1 and RA1-like proteins also carry an additional putative EAR motif in between. So far, experiments on the role of the third putative EAR motif observed in RA1 and RA1-like were not carried out yet. Then, RA1 sequences of the Andropogoneae members may have up to three putative EAR motifs. The phylogeny presented here supports the hypothesis of an increment in the number of putative EAR motifs during the evolution of SUP/RA1. Interestingly, it is well documented that a greater number of Leu along the EAR, a greater number of EAR motifs along the amino acid sequences, and regions that border EAR motifs are equally important for stabilizing binding to repressor proteins such as TPL [45]. These results suggest that, given the differences in the number and conservation of Leu residues of the EAR motifs, RA1 may have evolved towards an increased strength of repression activity through more stable binding to its co-repressor counterpart. 

#### 4.1.2. Changes in Secondary Structures

Studies on how folding information is distributed along a protein sequence provide information on the formation of different secondary structures, such as α-helices and β-sheets, and on binding properties [60]. The secondary structure predictions carried out in this work suggest that SUP, RA1, and RA1-like mainly differ towards the C-terminal. Such differences are correlated with the presence of one to three Motif 2, identified here as putative EAR motifs. Interestingly, EAR motifs were, previously, described as important motifs for the interaction with co-repressors [2,45,59]. 

Most zinc finger proteins of plants bind DNA via a short α-helix containing the highly conserved QALGGH sequence [61,62]. Interestingly, RA1 orthologs are characterized by having the QGLGGH sequence instead [2]. It has been suggested that Gly may act as a helix-relaxing residue that confers unique functional attributes [2]. Our analysis identified that the SUP zinc finger domain secondary structure consists of a very well-defined ββα motif, as was experimentally determined [33]. The same structure was predicted for the RA1 zinc finger domain, suggesting that RA1 binds DNA in a similar way to all other C2H2 zinc finger proteins structurally characterized [33,62,63,64,65,66,67,68,69,70]. The RMSF studies presented in this work showed that the change of Ala to Gly in the QALGGH motif of the RA1 zinc finger does not affect the mobility of the helix region, as was suggested in a previous work [2]. 

On the other hand, the zinc finger is one of the major structural motifs involved in eukaryotic protein–DNA interaction [60,71]. Structural studies on the C2H2 zinc finger have revealed that three positions (determinant residues) in the helical region of the zinc finger participate in major interactions with sequences in target DNA. In animals, the three amino acid residues of the finger that specifically make contacts with the DNA bases occupy the helix positions +2 (Arg), +3 (Asn), and +6 (Arg), and in plants, two of them correspond to the sequence QALGGH [55,62,72,73,74,75,76]. In this context, in SUP, the QALGGH sequence occupies positions 2–7 of the helix; that is, it includes all three residues (+2, +3, and +6) [33]. In SUP, position 2 is occupied by a Gln (Q) residue, position 3 by an Ala (A), and position 6 by a Gly (G) residue [33]. Even though Q and more infrequently A residues are reported to act as base determinants, it is difficult to conceive that G can bind a base of the nucleotide due to the lack of a side chain [33]. Indeed, it has been proposed that the binding of SUP is performed through residues at relative positions −1 (S), 2 (Q), and 3 (A) [33]. The G residues at relative positions 5 and 6 could allow the helix to draw closer to the DNA, and the residues at the C-terminal of the zinc finger helix relative positions 9 (N), 10 (V), and 12 (R), may bind the DNA, as in the case of EPF2-5 and EPF2-7 of petunia [77]. For the case of the RA1 residue at relative position, 3 is a G; therefore. the proposal of positions -1, 2, and 3 as base determinants can be ruled out. Nevertheless, it is still possible that G plays a passive role in the approximation of the α-helix to the major groove of DNA, allowing other determinant residues upstream in the structure to bind the DNA bases [33]. In the same way, taking the alternative proposal of Isernia et al. [33], the determinant residues would be 9 (N), 10 (I), and 12 (R) near the zinc finger α-helix C-terminal. The variability of residues in this zinc finger sequence among zinc finger proteins could be related to specificities for different and unique base triplet sequences, generating a huge diversity of target sequences [62,76]. To explore these possibilities, additional experiments are needed that, however, are beyond the scope of this work.

#### 4.1.3. Changes in Promoter Binding Properties

Regulatory motifs in promoter regions serve as recognition sites for TFs that promote the initiation of transcription as well as specific regulation of gene expression [78]. In general, most of the *cis*-acting elements identified in all promoters suggest that these proteins are related to plant growth and development but via different pathways or interacting with diverse partners.

The analysis of regulatory elements in the potential promoters of RA1 and RA1-like genes identified binding motifs for TFs involved in seed germination (ABI3, [79]; ABI5, [80]; FUS3, [81]), plant development (BPC5, BPC6, [82]; TB1, [83]; ARF4, [84]; ARF16, [85]; RA1, [2]; SMZ, TGA9, [86]; bHLH130, [87]; SPL14, [88]; ATHB12, [89]; AGL27, [90]; AGL42, [91]; CDF5, [92]; ARALYDRAFT_897773, also known as TCP4, [93]; ARALYDRAFT_496250, also known as TCP5, [94]; GRF9, [95]; ANL2, [96]; PLT1, [97]; HHO3, [98]; ABF2, [99]; DOF3.6, [100]), response to abiotic stress (DREB1E, [101]; ERF008, [102]; ERF055, [103]; ERF115, [104]; NAC020, [105]; NAC045, [106]; NAC092, [107]; OsRR22, [108]), and response to biotic stress (WRKY62, [109]; WRKY75, [110]; MYB73, [111]). The partial conservation of *RA1* and *RA1-like* promoters suggests that, after duplication, the genes have partially retained the ancestral upstream network and also acquired diverse regulatory pathways. It would be interesting to investigate how changes in the promoter region correspond to motif diversification and protein structure.

The conserved promoter sites found in the *RA1* sequences in this study are consistent with the findings of Strable et al. [6]. The authors identified several highly conserved motifs in the *RA1* promoter sequences of Andropogoneae species, such as ARFs-motifs, which were also identified in the present study. Also, our findings support the hypothesis that RA1 plays a role in the transition to flowering, the integration of both developmental and environmental cues [5], as well as the positive or negative regulation of gene expression in specific organs and at certain stages of development [53]. Additionally, our results suggest that these sites may also be involved in the regulation of hormonal pathways. In line with this, previous RNA-seq and Chip-seq studies have also indicated that RA1 may be involved in the biosynthesis and signaling of gibberellic acid and linked to auxin pathways [5]. 

## 5. Conclusions

To link the fragmented and sometimes conflicting knowledge on the evolution and functionality of SUP/RA1 proteins, in this work, we present (1) a solid phylogenetic framework to understand the origin and evolution of RA1 in embryophytes, (2) a detailed SUP/RA1 homology clustering among model and non-model embryophyte species, and (3) new insights on the *RA1* gene features and protein structure evolution. 

The phylogenetic reconstruction presented in this work suggests that RA1 arose from two successive SUP duplications during the diversification of grass species. This gave rise to three different grass sequence lineages, namely RA1-like A, RA1-like B, and RA1, most of which have unknown functions. The phylogenetic distance and duplication events that separated SUP and RA1 may explain the different roles reported for these proteins. Interestingly, the genomes of most studied species have retained RA1 and RA1-like proteins, underscoring their functional significance.

Our findings indicate that RA1 and RA1-like play roles in the nucleus despite lacking a traditional nuclear localization signal. Here, we report that copies diversified their coding region and, with it, their protein structure, suggesting different patterns of DNA binding and protein–protein interaction. In addition, each of the retained copies diversified regulatory elements along their promoter regions, indicating differences in their upstream regulation. Overall, our analyses suggest that RA1 and RA1-like are likely involved in different pathways of plant growth and development. Therefore, the evidence indicates that the *RA1* and *RA1-like* gene families in grasses underwent subfunctionalization and neofunctionalization enabled by gene duplication.

## Data Availability

The data presented in this study are openly available in Mendeley Digital Repository at https://doi.org/10.17632/m45fk8hxs4.1, accessed on 8 February 2024.

## Supplementary Materials

The following supporting information can be downloaded at: https://www.mdpi.com/article/10.3390/biom14050550/s1, Figure S1: Multiple sequence alignment of 73 C2H2 zinc finger amino acid sequences from 48 embryophyte species; Figure S2: Bayesian consensus tree of RA1 TF in grasses; Figure S3: Bayesian consensus tree of RA1 and RA1-like B TFs in grasses; Figure S4: Scheme representing the presence or absence of RA1 and RA1-like proteins in grass species; Figure S5: Chromosome synteny between *A. thaliana* and *Z. mays*; Figure S6: Chromosome synteny between *O. sativa*, *Z. mays*, and *S. viridis*; Figure S7: Schematic distribution of conserved motifs in RA1 and RA1-like proteins; Figure S8: Motif consensus sequences identified by MEME; Figure S9: Multiple sequence alignment of 35 C2H2 zinc finger amino acid sequences from 16 grass species; Figure S10: Proteins conformational plasticity; Figure S11: Zinc finger dynamic simulation; Table S1: Protein data information from embryophyte genomes; Table S2: Primers and enzymes used in each genetic construction; Table S3: Functional characterized proteins information; Table S4: Proteins conformational plasticity and secondary structures; Table S5: *Cis*-acting regulatory elements analysis; Table S6: TF motifs associated a different biological processes.
