# Supplementary material for: Molecular Evolution of RAMOSA1 (RA1) in Land Plants
Source: Biomolecules. 2024 May 3;14(5):550. doi: 10.3390/biom14050550 (PMC11117814; doi:10.3390/biom14050550)
Supplement: Supplementary file 1 [file biomolecules-14-00550-s001.zip › Supplementary Materials V2/Figure S10.pdf]

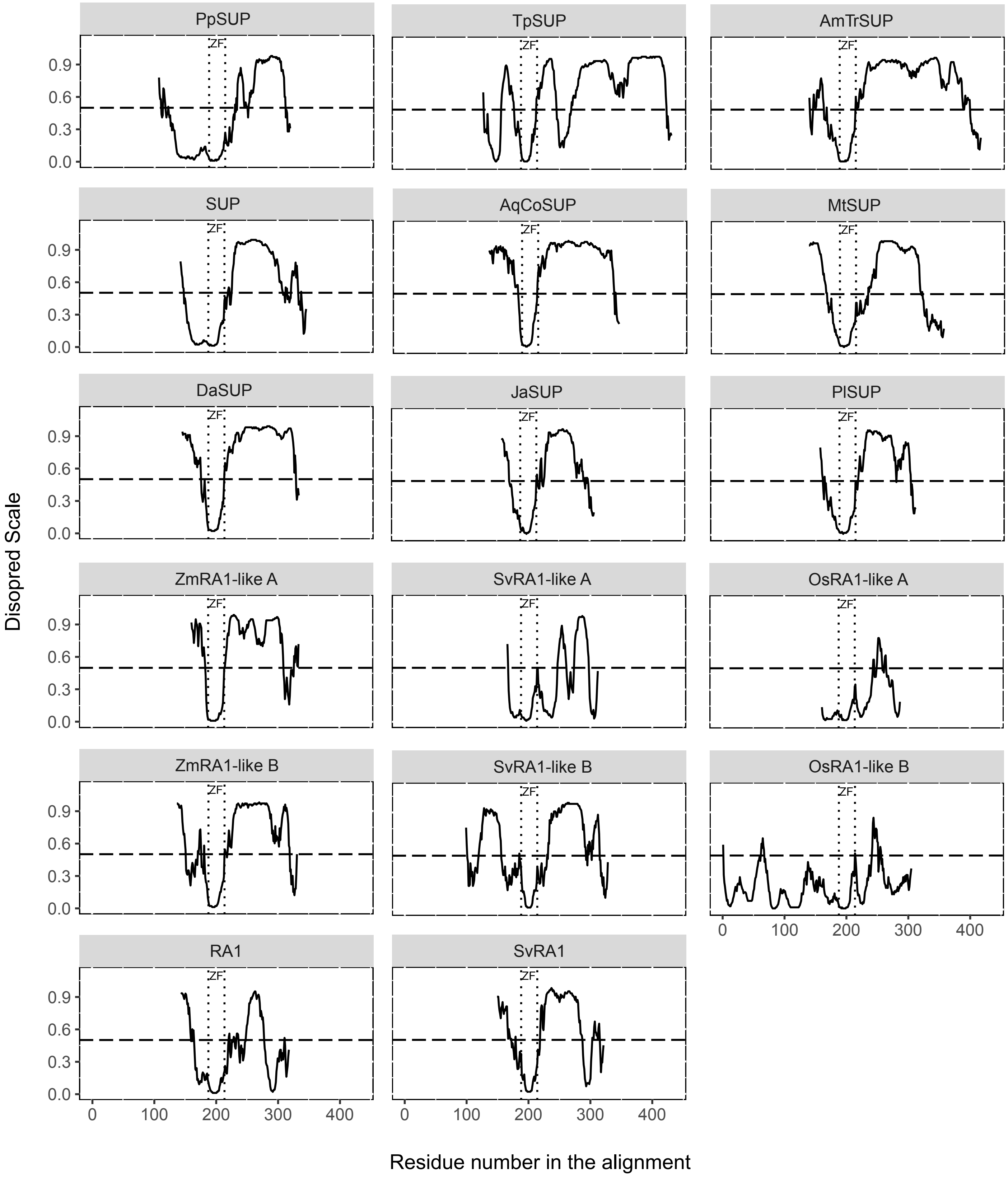

Figure S10. Proteins conformational plasticity. Relative disorder levels of the structures are measured in a range of 0 to 1.0, following the algorithms used by DisoPred 3.1 server. Levels above 0.5 (dashed line) indicate disordered regions. The segment corresponding to the zinc finger (ZF) domain is marked in the figure.
