# Supplementary material for: Molecular Evolution of RAMOSA1 (RA1) in Land Plants
Source: Biomolecules. 2024 May 3;14(5):550. doi: 10.3390/biom14050550 (PMC11117814; doi:10.3390/biom14050550)
Supplement: Supplementary file 1 [file biomolecules-14-00550-s001.zip › Supplementary Materials V2/Figure S11.pdf]

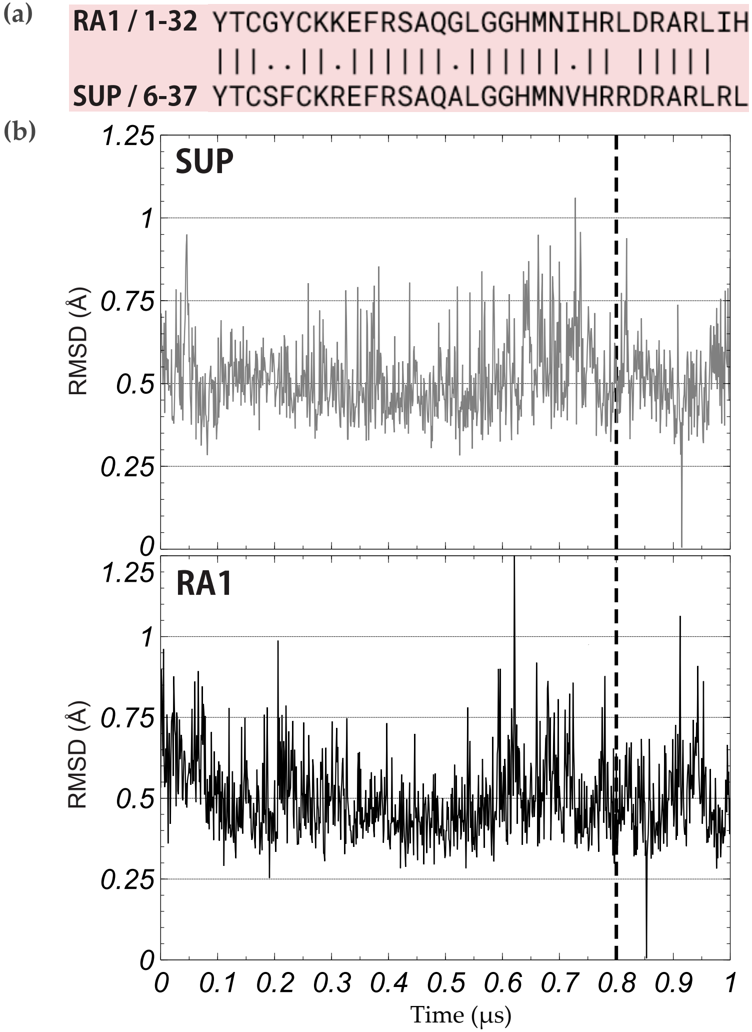

Figure S11. Zinc finger dynamic simulation. (a) Local sequence alignment of RA1 and SUP zinc finger domain as determined by Smith-Waterman algorithm (residue identity 75%). (b) Time evolution of the RMSD of the backbone atoms of the RA1 (above) and SUP (below) peptides in the simulated interval 0.0 to 1.0 μs. The structure with the smallest RMSD in the interval 0.0 to 1.0 μs of each simulation has been used as reference for fitting the structures of each peptide. The vertical gray dashed line at 0.8 μs marks the time that has been assumed as the beginning of the thermodynamic equilibrium interval for properties calculations.
