# Supplementary material for: Molecular Evolution of RAMOSA1 (RA1) in Land Plants
Source: Biomolecules. 2024 May 3;14(5):550. doi: 10.3390/biom14050550 (PMC11117814; doi:10.3390/biom14050550)
Supplement: Supplementary file 1 [file biomolecules-14-00550-s001.zip › Supplementary Materials V2/Figure S3.pdf]

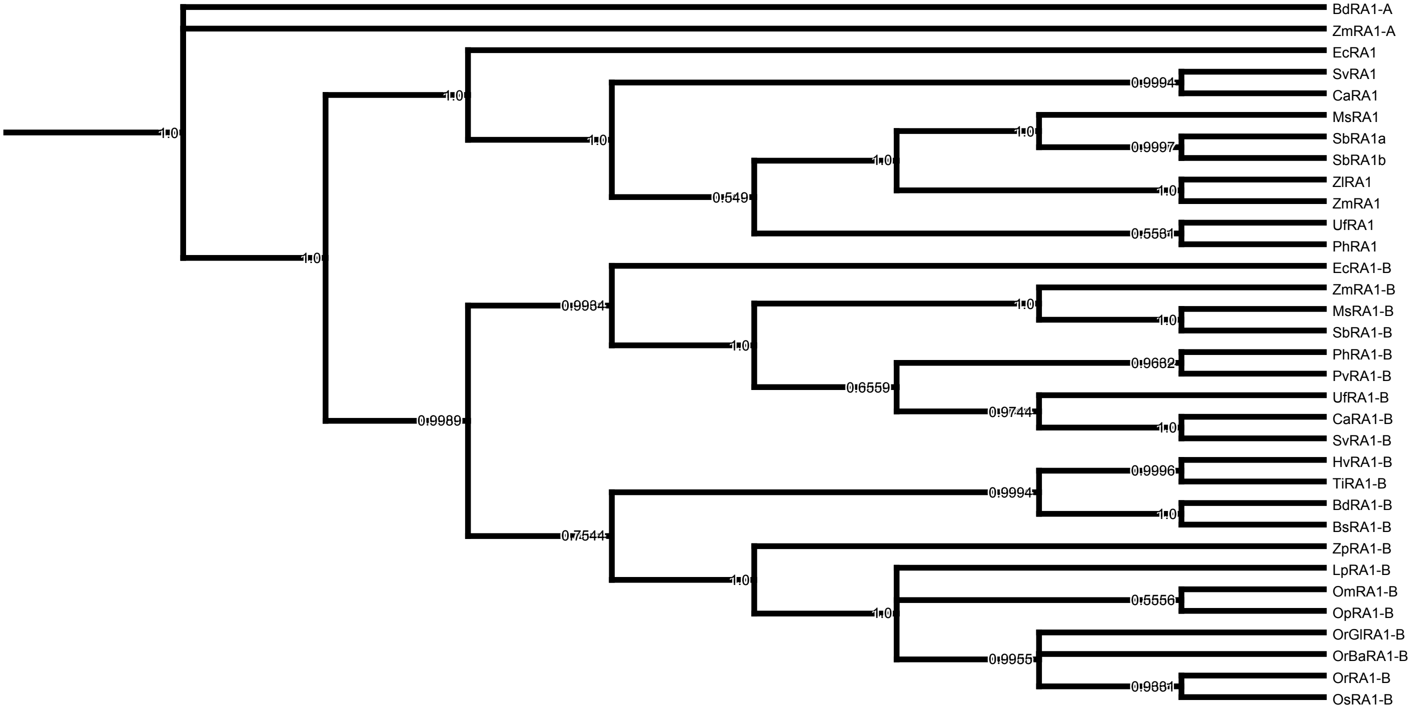

Figure S3. Resolved phylogenetic tree between RA1 and RA1-like B. Majority rule consensus tree (N=22502 trees) of RA1 and RA1-like B TFs in grasses generated by Bayesian inference using 33 amino acid sequences. BdRA1-A and ZmRA1-A were used as outgroups.
