# Supplementary material for: Molecular Evolution of RAMOSA1 (RA1) in Land Plants
Source: Biomolecules. 2024 May 3;14(5):550. doi: 10.3390/biom14050550 (PMC11117814; doi:10.3390/biom14050550)
Supplement: Supplementary file 1 [file biomolecules-14-00550-s001.zip › Supplementary Materials V2/Figure S4.pdf]

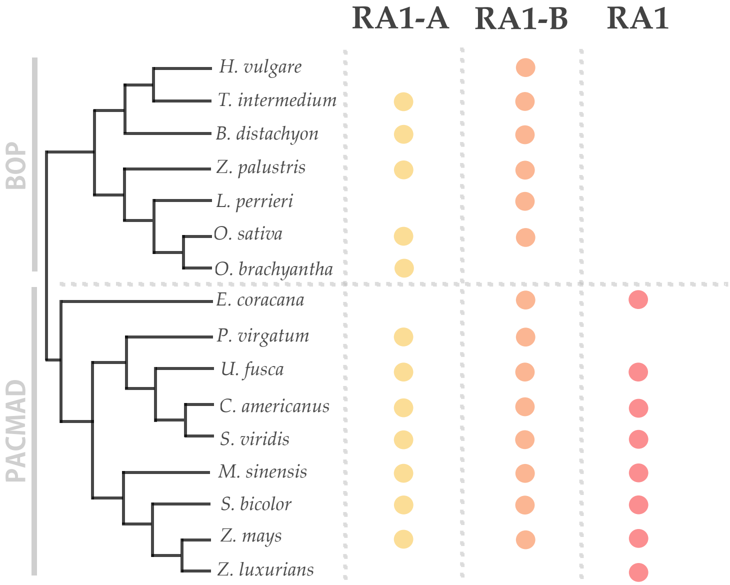

Figure S4. Scheme representing the presence or absence of RA1 and RA1-like proteins in grass species. Colored circle represents the presence of the protein.
