# Supplementary material for: Molecular Evolution of RAMOSA1 (RA1) in Land Plants
Source: Biomolecules. 2024 May 3;14(5):550. doi: 10.3390/biom14050550 (PMC11117814; doi:10.3390/biom14050550)
Supplement: Supplementary file 1 [file biomolecules-14-00550-s001.zip › Supplementary Materials V2/Figure S5.pdf]

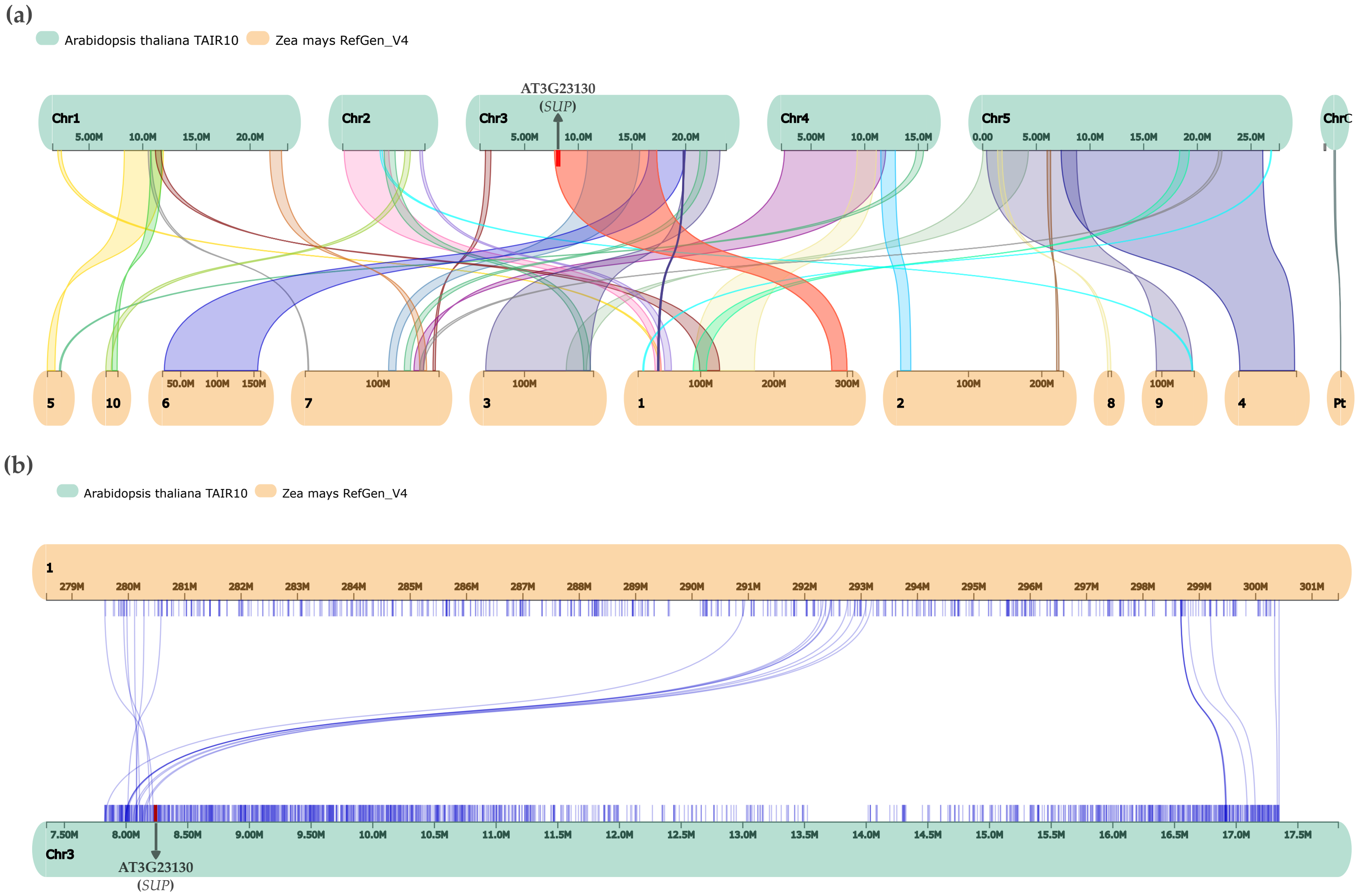

Figure S5. Chromosome synteny between *A. thaliana* and *Z. mays*. (a) Analysis of synteny between genomes. Colored lines link collinearity blocks that represent syntenic regions identified by GENESPACE. (b) Zoom in of gene collinearity between chromosome 3 of *A. thaliana* and chromosome 1 of *Z. mays*.
