# Supplementary material for: Molecular Evolution of RAMOSA1 (RA1) in Land Plants
Source: Biomolecules. 2024 May 3;14(5):550. doi: 10.3390/biom14050550 (PMC11117814; doi:10.3390/biom14050550)
Supplement: Supplementary file 1 [file biomolecules-14-00550-s001.zip › Supplementary Materials V2/Figure S6.pdf]

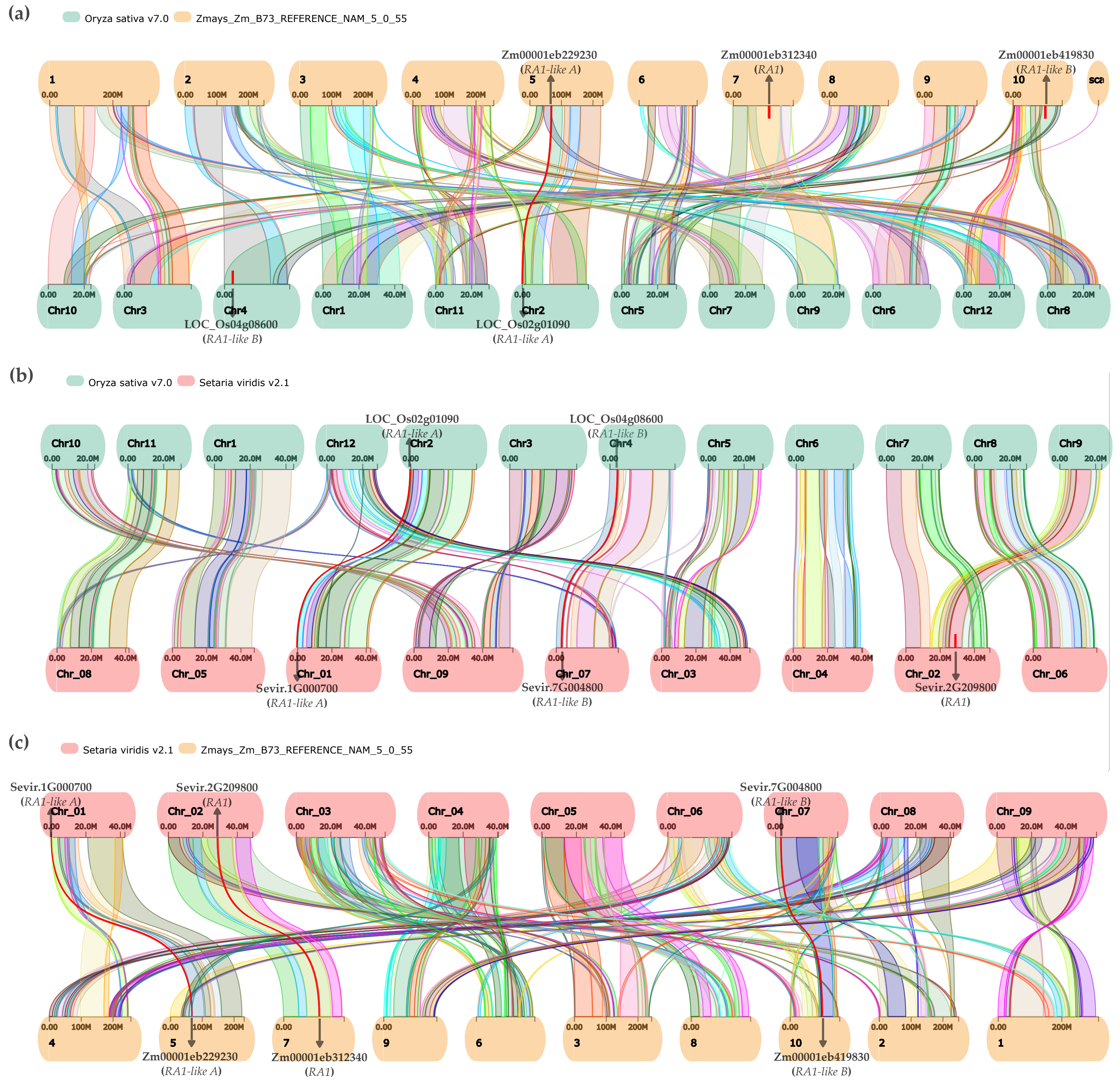

Figure S6. Chromosome synteny between *O. sativa, Z. mays,* and *S. viridis*. (a) Analysis of synteny between *O. sativa* and *Z. mays* genomes. Colored lines link collinearity blocks that represent syntenic regions identified by GENESPACE. (b) Analysis of synteny between *O. sativa* and *S. viridis* genomes. Colored lines link collinearity blocks that represent syntenic regions identified by GENESPACE. (c) Analysis of synteny between *S. viridis* and *Z. mays* genomes. Colored lines link collinearity blocks that represent syntenic regions identified by GENESPACE.
