# Supplementary material for: Molecular Evolution of RAMOSA1 (RA1) in Land Plants
Source: Biomolecules. 2024 May 3;14(5):550. doi: 10.3390/biom14050550 (PMC11117814; doi:10.3390/biom14050550)
Supplement: Supplementary file 1 [file biomolecules-14-00550-s001.zip › Supplementary Materials V2/Figure S8.pdf]

Symbol	Motif	Logo	Consensus sequence	Width (aa)	E-value
	Motif 1	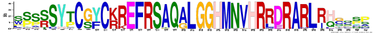	SSSSSYTCGYCKREFRSAQALGGHMNVHRRDRARLRHGQSP	41	9.9e-1061
	Motif 2	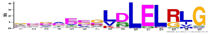	GDGAEEGLDLELRLG	15	1.2e-282
	Motif 3	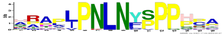	ARAPJPNLNYSPPHPA	16	1.5e-134
	Motif 4	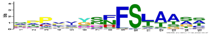	APPVVYSFFSLAASA	15	1.3e-080
	Motif 5	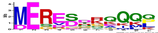	MERESDKQQQQ	11	6.2e-032
	Motif 6	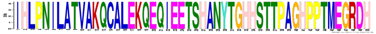	IHLPNILATVAKQCALEKQEQIEETSHANYTGHHSTTPAGHPPTMEGRDH	50	2.7e021
	Motif 7	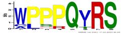	WPPPQVRS	8	2.3e-014
	Motif 8	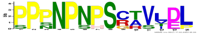	PPPNPNPSCTVLDL	14	1.5e-009
	Motif 9	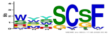	WYYSCSF	7	3.2e-005
	Motif 10	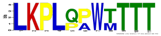	LKPLQPWTTTT	11	1.3e-004
	Motif 11	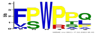	FPWPPQ	6	1.6e-004
	Motif 12	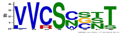	VVCSCSST	8	6.2e-003
	Motif 13	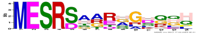	MESRSAARAGDQQH	14	9.6e-004
	Motif 14	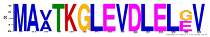	MAVTKGLEVDLELEV	15	4.6e-001
	Motif 15	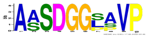	AASDGGLAVP	10	6.4e+000

Figure S8. Motif consensus sequences identified by MEME based on 35 RA1 and RA1-like sequences identified for 16 grass species. Motif 1 represents the conserved zinc finger domain. Motif 2 represents the conserved EAR motif. Motifs presence and distribution along each of the sequences is presented in Figure S7.
