# Supplementary material for: Molecular Evolution of RAMOSA1 (RA1) in Land Plants
Source: Biomolecules. 2024 May 3;14(5):550. doi: 10.3390/biom14050550 (PMC11117814; doi:10.3390/biom14050550)
Supplement: Supplementary file 1 [file biomolecules-14-00550-s001.zip › Supplementary Materials V2/Figure S9.pdf]

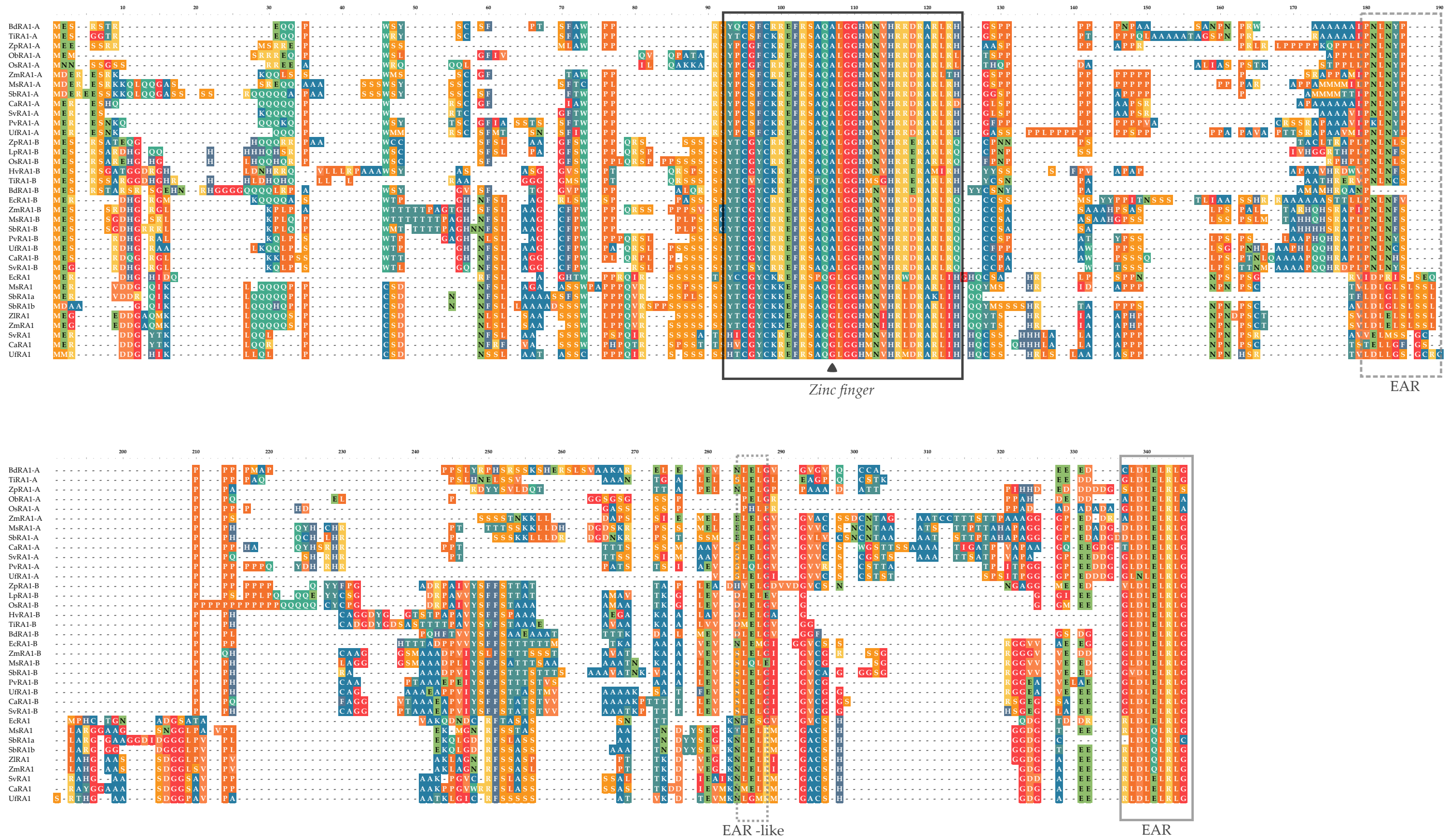

Figure S9. Multiple sequence alignment of 35 C2H2 zinc finger amino acid sequences from 16 grass species. Black box indicates the zinc finger domain (Motif 1). Grey boxes indicate the putative EAR motifs (Motif 2). Dashed gray box indicates the putative EAR motif partially conserved among these proteins. Dotted gray box indicates a motif composed by only two Leu, similar to an EAR motif (EAR-like). Solid gray box indicates the EAR motif towards the C-terminal. Black arrowhead indicates the amino acid position changed (A -> G) in the zinc finger domain.
