# Supplementary figures and images for: Molecular Evolution of RAMOSA1 (RA1) in Land Plants
Source: Biomolecules. 2024 May 3;14(5):550. doi: 10.3390/biom14050550 (PMC11117814; doi:10.3390/biom14050550)

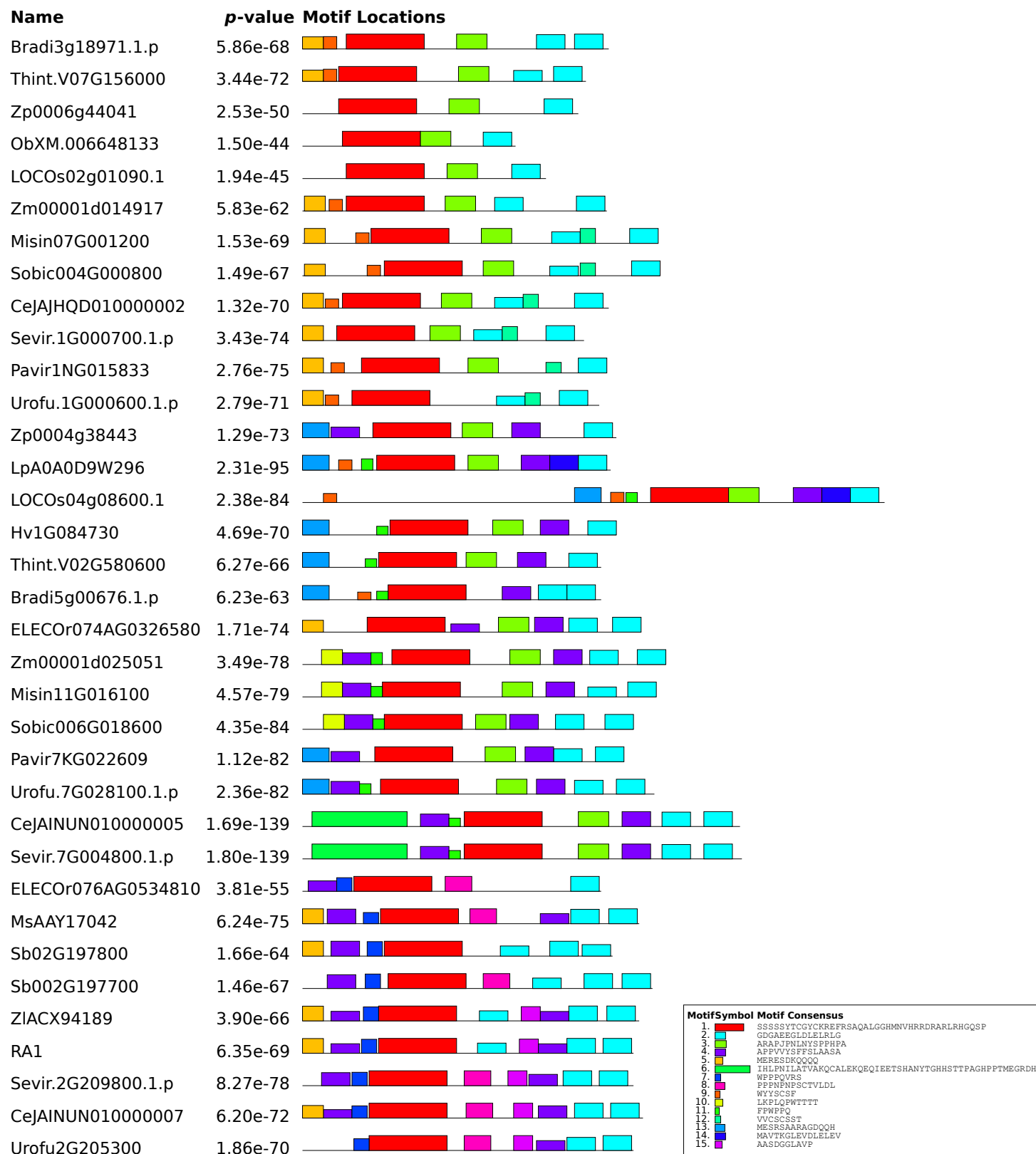

Supplement: Supplementary file 1 [file biomolecules-14-00550-s001.zip › Supplementary Materials V2/Figure S7.pdf]
