# Supplementary material for: Molecular Evolution of RAMOSA1 (RA1) in Land Plants
Source: Biomolecules. 2024 May 3;14(5):550. doi: 10.3390/biom14050550 (PMC11117814; doi:10.3390/biom14050550)
Supplement: Supplementary file 1 [file Figure S2.pdf › Supplementary Materials V2/Figure S2.pdf]

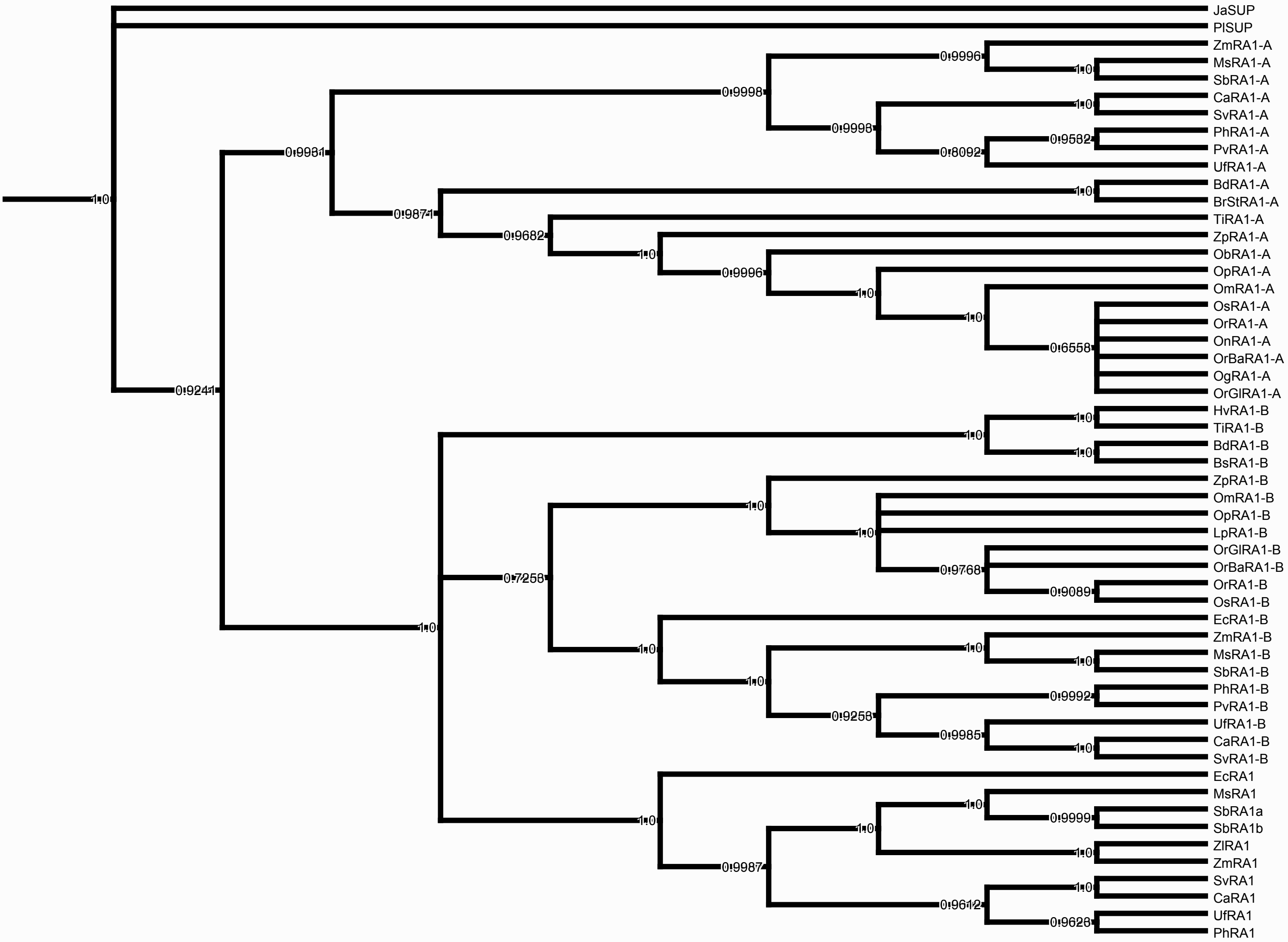

Figure S2. RA1 evolution in grasses. Majority rule consensus tree (N=22502 trees) of RA1 TF in grasses generated by Bayesian inference using 54 amino acid sequences. JaSUP was used as outgroup.
